# Oral Health, Polypharmacy and Nutritional Status in Institutionalized Dementia Patients: A Multicenter Cross-Sectional Study

**DOI:** 10.3390/biomedicines14071476

**Published:** 2026-06-29

**Authors:** Joana Pombo-Lopes, Diogo Sousa-Catita, Paulo Mascarenhas, Jorge Fonseca, José Grillo-Evangelista

**Affiliations:** 1 Clinical Research Unit (CRU), Centro de Investigação Interdisciplinar Egas Moniz (CiiEM), Egas Moniz Cooperativa de Ensino Superior, 2829-511 Almada, Portugal; diogo.rsc2@gmail.com (D.S.-C.); pmascarenhas@egasmoniz.edu.pt (P.M.); jfonseca@egasmoniz.edu.pt (J.F.); jgrillo@egasmoniz.edu.pt (J.G.-E.); 2 Aging Lab, Egas Moniz Center for Interdisciplinary Research (CiiEM), Egas Moniz School of Health & Science, 2829-511 Almada, Portugal; 3 Morphology Lab, Egas Moniz Center for Interdisciplinary Research (CiiEM), Egas Moniz School of Health & Science, 2829-511 Almada, Portugal; 4 Residências Montepio, Serviços de Saúde, SA., 1600-131 Lisbon, Portugal; 5 GENE—Artificial Nutrition Team, Department of Gastroenterology, Hospital Garcia de Orta, 2805-267 Almada, Portugal

**Keywords:** dementia, oral health, nutritional status, edentulism, polypharmacy

## Abstract

**Background:** As the population ages, dementia poses a critical public health challenge. This study examined the oral health and nutritional status of institutionalized Portuguese adults with dementia, exploring their interrelated predictors. **Methods:** This multicenter, cross-sectional study assessed institutionalized patients using the Decayed, Missing, and Filled Teeth (DMFT) index, posterior functional units (PFUs), plaque (PI) and gingival (GI) indices, the Short Xerostomia Inventory (SXI-5), and the Mini Nutritional Assessment (MNA). DMFT was modeled using multivariable ordinary least squares (OLS) regression for demographic and clinical predictors and separate negative binomial models for medication-related predictors. Other outcomes were analyzed using outcome-specific multivariable models. **Results:** The study included 71 participants (mean age: 82.5 ± 6.9 years). A high dental disease burden (mean DMFT score of 24.3 ± 7.5) was observed, independently predicted by advanced age (β = 0.48, *p* = 0.002) and residence in public long-term care units (LTCUs) (β = 6.65, *p* = 0.001). Total edentulism affected 28.2% of the sample. Polypharmacy emerged as a significant predictor of tooth loss; each additional medication was associated with an 18% decrease in the likelihood of retaining natural teeth (OR = 0.82, *p* = 0.008). Higher cognitive decline (GDS) was associated with increased plaque (*p* = 0.043), and modified-texture diets were associated with lower plaque levels (β = −0.64, *p* = 0.021). The mean MNA score (16.9 ± 3.8) indicated a high risk of malnutrition, with a trend toward lower gingival inflammation with better nutritional status (*p* = 0.061). **Conclusions:** Institutionalized dementia patients face severe oral and nutritional risks associated with age, polypharmacy and institutional environment. This emphasizes the need for multidisciplinary protocols and caregiver training.

## 1. Introduction

Across most developed nations, the population is aging. According to Eurostat, the European Union (EU) population is projected to increase from 446.7 million in 2022 to 453.3 million in 2026 (+1.5%), of which 21.1% are aged 65 or older [1]. Life expectancy in the EU has also been rising again, reaching 81.4 years in 2023, surpassing pre-pandemic levels [2].

Dementia, driven by demographic aging, is an increasingly critical public health challenge, and Portugal is no exception [3]. According to Alzheimer Europe, an estimated 8,885,101 people were living with dementia in the EU-28 in 2018, corresponding to a prevalence of approximately 1.73% of the population [4]. In Portugal, the same report estimated a prevalence of 1.88%, equivalent to around 193,516 persons in 2018 [4]. Projections suggest that the number of persons with dementia in Portugal could rise to 346,905 by 2050, increasing the prevalence to around 3.82%, largely due to growth in older age groups (especially those over 70 and 85 years old) [4]. Previous studies have also estimated that the prevalence of dementia among Portuguese citizens aged 60 or older is approximately 5.9%, equating to more than 160,000 individuals [5].

Adults with dementia face oral health challenges due to impaired self-care and hygiene maintenance, decreased access to dental care, and the cumulative effects of these factors over time [6,7,8]. Consequently, people with dementia exhibit a wide range of oral health issues, including elevated plaque levels, increased gingival bleeding, higher rates of periodontal disease, and greater levels of untreated tooth decay. They also tend to have fewer remaining teeth, more soft tissue lesions, xerostomia (dry mouth subjective sensation), and other oral mucosal problems [9]. The quality of life of people with dementia is greatly affected by their oral health [6,8,10,11,12].

The nutritional status of individuals with dementia is crucial for predicting their clinical outcome, given the well-established link between aging, cognitive decline and malnutrition, which often results in a poor prognosis [13,14]. Research involving institutionalized elderly people has highlighted the high prevalence of malnutrition and some difficulties in using nutrition assessment tools [13,15]. Poor oral health, including tooth loss, reduced chewing ability, dry mouth or oral lesions, can restrict the range of foods consumed, particularly harder, fiber-rich foods such as fruit, vegetables and meat [16,17]. Over time, these dietary restrictions can lead to protein-energy malnutrition. Furthermore, dysphagia, a usual symptom of neurodegenerative decline, leads to reduced nutrient intake, creating a cycle of deteriorating health that requires clinical intervention [17]. Although the use of enteral feeding in dementia patients is debated, evidence suggests that interventions such as percutaneous endoscopic gastrostomy (PEG) can stabilize or improve nutritional markers and positively impact survival outcomes for carefully selected patients with strong social support [13,14].

Although the negative impact of dementia on oral health is well documented, with a marked decline in self-care and hygiene as the neurodegenerative disease progresses, there is still a significant lack of robust data on the interaction between specific polypharmacy and nutritional status in institutionalized patients. The high prevalence of polypharmacy in these patients, particularly the continued use of antipsychotics and antidepressants, is associated with side effects such as xerostomia and accelerated tooth loss, which severely compromise chewing ability. This, in turn, acts as a barrier to the intake of fresh and fibrous foods, exacerbating the already high prevalence of malnutrition in this population. Thus, national literature lacks integrated evidence crossing these different biological dimensions within the same sample group, making it difficult to define public health policies and multidisciplinary care protocols adapted to the Portuguese context. The present study aimed to assess the oral health status of institutionalized adults with dementia in Portugal and explore its relationship with nutritional status. We hypothesized that oral health deficits, such as tooth loss and poor hygiene, could be associated with poorer nutritional status and polypharmacy.

## 2. Materials and Methods

### 2.1. Study Design

The present study was designed as a cross-sectional study, adhering to the STROBE guidelines [18] and complying with the 2013 Declaration of Helsinki [19]. Formal approval of the study protocol was granted by both the ethics committees of the Egas Moniz School of Health and Science (approval 1183, 26 January 2023) and the Montepio Residences (21 November 2022). All patients or their legal representatives signed an informed consent form permitting the use of their anonymized dental and medical histories for research purposes.

### 2.2. Study Population

Participants were recruited using a convenience sampling approach. All adults diagnosed with dementia at one of the four rehabilitation centers of the Montepio Residences in Lisbon (Portugal) during the study period (2023–2025) were invited to participate, and no further specific selection criteria were applied. These units offer two types of care settings: (1) private residential wings for long-term residents, and (2) public Long-Term Care Units (LTCUs), which provide temporary clinical monitoring and rehabilitation as part of the Portuguese National Network for Integrated Continuous Care. Exclusion criteria included: (i) lack of informed consent from the patient or legal representative; (ii) clinical instability or severe frailty that prevented a safe oral examination; and (iii) inability to cooperate with the assessment (e.g., persistent unresponsiveness or refusal to open the mouth).

### 2.3. Assessed Parameters

Sociodemographic and medical characteristics were obtained from patients’ medical charts. Sociodemographic variables included sex, age, marital status, and level of education. Medical variables included the presence of comorbidities (diabetes mellitus, stroke, hypertension, or others), tobacco use and the number of medications used per day. Polypharmacy was defined as the regular use of five or more medications per day. This specific threshold was selected because it is the most prevalent numerical definition in geriatric research, utilized by approximately 46.4% of studies in the field [20]. It is also the standard adopted by the World Health Organization (WHO) for monitoring medication safety risks, such as prescribing errors and adverse drug events [21]. Cognitive impairment was also assessed based on dementia diagnoses recorded in patients’ medical charts by their attending physicians. Cognitive and functional deterioration were characterized using the Global Deterioration Scale (GDS), a seven-stage clinical scale designed to describe the progression of cognitive decline associated with primary degenerative dementia, particularly Alzheimer’s disease. It ranges from stage 1 (no cognitive decline) to stage 7 (very severe cognitive decline) [22].

Oral characteristics were recorded during a clinical oral examination by a single dentist (J.P.L.), in accordance with World Health Organization (WHO) recommendations [23]. Caries experience was assessed using the Decayed, Missing, Filled Teeth (DMFT) index, following WHO methodology. The DMFT index ranges from 0 to 32 (including third molars), with higher values indicating a greater lifetime exposure to dental caries [23,24]. Because of the cross-sectional nature of the study and the absence of long-term dental records, the “M” component of DMFT represents total tooth loss, not just teeth lost due to dental caries. For this cohort, many of whom have aphasia or severe memory loss, it was clinically unfeasible to differentiate between teeth lost due to caries, periodontal disease, or prosthetic extractions. Thus, the DMFT index in this study serves as a proxy for the total accumulated burden of oral disease throughout the participants’ lives. Periodontal status was quantified using the Plaque Index (PI) and the Gingival Index (GI), with scores ranging from 0 to 3 based on the severity of plaque accumulation and gingival inflammation [25]. The prosthetic status of each jaw was recorded as follows: no denture; partial removable denture; full removable denture; fixed denture; or a combination of these. Stability, retention, structural defects, and hygiene methods of existing dentures were assessed. A prosthesis was deemed necessary if the existing dentures were clinically unsatisfactory or absent. Additionally, the need for dental treatment was assessed based on clinical judgment according to WHO Oral Health Surveys criteria, and categorized as routine/preventive care, urgent care (due to pain or infection), or immediate care [23]. Posterior Functional Units (PFUs) were defined as pairs of posterior antagonist teeth (natural or prosthetic) that establish functional contact during chewing. To determine PFUs counts, patients chewed on 200 µm articulating paper, and colored marks on the inferior molars and premolars were recorded [26]. For patients who use dentures irregularly, the assessment only considered natural units to reflect their actual functional capacity. Subjective symptoms were evaluated using questionnaires. Self-reported oral pain was recorded as present or absent based on the occurrence of pain or discomfort in the teeth or mouth during the previous 12 months. Xerostomia was assessed using the Short Xerostomia Inventory (SXI-5), a 5-item summated scale with items rated from 1 (never) to 3 (frequently), where higher total scores indicate greater perceived severity [27]. These assessments were only given to participants who could verbally communicate and follow simple instructions during the clinical examination, as shown in Table A1. Finally, although data on oral hygiene assistance and dental visit history were initially collected via patient questionnaires, these variables were excluded from the final multivariable models to prevent significant recall bias.

A detailed analysis of daily medications was performed to evaluate the impact of pharmacological therapy on oral health. Anticholinergic burden was quantified using a validated electronic calculator (https://www.acbcalc.com/), which integrates two of the most reliable and valid scoring systems in the literature: the German Anticholinergic Burden Score [28] and the Anticholinergic Cognitive Burden (ACB) Scale [29]. Individual drug scores, ranging from 0 (no effect) to 3 (markedly anticholinergic), were summed to calculate the total cumulative burden. This burden was analyzed as a continuous variable and in three categories: 0, 1–2, and ≥3. Although the scale was initially designed to predict cognitive outcomes, it is a validated tool for estimating peripheral adverse effects, such as drug-induced xerostomia. Additionally, binary indicators were created for high-risk classes, including anticoagulants, antiplatelets, calcium channel blockers and diuretics.

The Mini Nutritional Assessment (MNA) was used to evaluate the participants’ nutritional status by a single registered dietitian (DC). MNA has been specifically validated for use with elderly populations and has been shown to be more useful than more general instruments for detecting malnutrition risk [16]. The calculation is based on evaluating six different areas of concern, including anthropometric parameters such as Body Mass Index (BMI), unintentional weight loss, and a neuropsychological evaluation (Item E) that classifies the patient’s mental state as normal, mild dementia, or severe dementia [16,30]. For bedridden patients or those with reduced mobility, BMI can be accurately estimated through regression equations using the mid-upper arm circumference (MUAC) [16,30] and regression equations described by Powell-Tuck and Hennessy [31], which provide a reliable estimation of BMI in bedridden patients [31,32]. A score of under 17 indicates malnutrition, a score of 17 to 23.5 indicates risk of malnutrition, and a score of 24 to 30 indicates normal nutritional status [30].

### 2.4. Statistical Analysis

Statistical analyses were performed using R software. Quantitative variables are reported as mean ± standard deviation (SD), and categorical variables as absolute and relative frequencies.

A complete-case approach was adopted for all multivariable analyses, resulting in different analytical samples across outcomes. DMFT and retained roots were analyzed in the full clinical sample (*n* = 71), whereas the analytical samples were smaller for oral pain (*n* = 56), xerostomia (SXI-5; *n* = 42), posterior functional units (PFUs; *n* = 65), and periodontal indices (PI and GI; *n* = 31). The reasons for these reductions are summarized in Table A1. Data imputation was not performed because missingness was considered missing not at random (MNAR), being related to dementia severity, communication barriers, and clinical non-cooperation.

Given the modest sample size and the number of candidate predictors, variable selection was guided by clinical plausibility, sample-size constraints, and the relative stability and magnitude of coefficients in ridge-penalized models. Ridge regression with L2 penalization was implemented as an auxiliary stability procedure using the glmnet package, with ten-fold cross-validation and the λ1se rule used to favor more parsimonious specifications. The final estimates reported in the tables were obtained from conventional outcome-specific regression models and therefore correspond to unpenalized estimates.

The modeling strategy was tailored to each outcome. The DMFT index was analyzed using two complementary approaches: a multivariable ordinary least squares (OLS) regression model for the main demographic and clinical predictors, and separate negative binomial regression models for medication-related predictors. OLS results are reported as unstandardized β coefficients, whereas negative binomial results are reported as incidence rate ratios (IRRs).

Retained roots were modeled using negative binomial regression. PFUs were modeled using a zero-inflated negative binomial model and restricted to participants with natural teeth or dental prostheses (*n* = 65), in order to avoid artificial floor effects from edentulous participants without prosthetic rehabilitation.

Continuous outcomes, including PI, GI and SXI-5, were analyzed using multivariable linear regression with heteroscedasticity-consistent HC3 robust standard errors. Total edentulism and self-reported oral pain were analyzed using binary logistic regression, with bias-reduced estimation considered when separation or numerical instability occurred. The number of natural teeth was analyzed using ordinal logistic regression.

Nutritional status was analyzed using the original MNA score. Statistical significance was set at a two-sided *p*-value < 0.05. Given the exploratory nature of the study and the reduced analytical samples for some outcomes, the results were interpreted cautiously as associations rather than causal effects.

## 3. Results

### 3.1. Participants’ Characteristics

The study population consisted of 71 elderly individuals. Some potential participants were primarily excluded due to a lack of informed consent from legal representatives, often associated with refusal to accept the dementia diagnosis. Others were excluded due to an inability to perform the oral examination, caused by patient non-cooperation, extreme frailty or unresponsiveness. Regarding their living environment, 32 participants (45.1%) resided in private nursing homes. In contrast, 39 (54.9%) were institutionalized in public Long-Term Care Units (LTCUs), which are part of the Portuguese National Network for Integrated Continuous Care. The mean age was 82.5 years (SD = 6.9, range 59–95), and 56.3% of participants were female. In terms of education and social background, most participants (62.0%) had only completed basic education (four years) and most were either married (45.1%) or widowed (38.0%).

Clinical findings revealed a high prevalence of polypharmacy, with an average of 8.9 medications per day (SD = 3.1, range 1–15) to manage common comorbidities. As expected, antidementia agents such as donepezil, memantine, and rivastigmine were the most prescribed, to 56 patients (78%). Other prevalent pharmaceutical classes identified include antipsychotics, specifically quetiapine (*n* = 27), and proton pump inhibitors (PPIs) for gastric protection (*n* = 26). Additionally, analgesics such as paracetamol (*n* = 23) and cardiovascular medications, particularly statins (*n* = 21), were also frequently prescribed. Lifestyle history revealed that 42.3% were non-smokers, and 26.8% were former smokers, some of whom smoked heavily, exceeding 40 cigarettes per day. However, only 2.8% were current smokers. Finally, a cognitive assessment using the Global Deterioration Scale (GDS) revealed that 26.8% presented with mild cognitive decline (GDS 3). However, a significant proportion of the cohort (66.2%) presented with more advanced stages of impairment, ranging from moderate to very severe decline (GDS 4–7). These sociodemographic and clinical characteristics are summarized in Table 1.

### 3.2. Oral Health and Nutritional Data

Table 2 summarizes the descriptive clinical and nutritional profile of the study participants. Although indices such as DMFT included the entire cohort (*n* = 71), the analytical samples for specific outcomes were smaller. These outcomes included oral pain (*n* = 56), xerostomia (*n* = 42), and periodontal indices (*n* = 31). Table A1 provides detailed reasons for these exclusions.

Participants’ oral hygiene habits were variable. Twenty-five participants (35.2%) reported brushing twice or more daily, while ten participants (14.1%) reported never brushing. The mean DMFT index was 24.31 (±7.48). Regarding the care environment and dental history, descriptive data showed that, of those capable of answering, only 12 reported receiving assistance with brushing their teeth, and only five admitted to having difficulty maintaining good oral hygiene. However, the majority (34/46) claimed to be autonomous in their daily routines. Professional dental visits were also infrequent. Thirty-two participants (45.1%) either could not recall their last appointment or said it had been over five years since their last visit. Twenty individuals (28.2%) were completely edentulous. Of those, 14 (19.7%) used prosthetic devices, while six (8.5%) were edentulous and did not receive prosthetic rehabilitation. Among the dentate group, 15 participants (21.1%) presented a prosthesis. A clinical evaluation of these prostheses revealed substantial structural and functional compromise. Half of the assessed devices (*n* = 12/24) were clinically maladapted. Five participants were excluded from this functional analysis because they owned prostheses but did not use them during the evaluation. This functional failure is closely linked to the devices’ extreme longevity. Of the 20 participants who could provide reliable information about the age of their prostheses, 70.0% (*n* = 14/20) had used at least one device for over a decade, with several exceeding 20 years of use. Nine participants were excluded from the temporal analysis due to severe communication barriers or an inability to recall. Similarly, 41.7% (*n* = 10/24) of the evaluated prostheses exhibited severe structural defects, primarily advanced wear, fissures in the base, and broken clasps. Prosthetic hygiene was unsatisfactory in most cases. Several participants merely rinsed their prostheses with water. Furthermore, nocturnal use habits were inconsistent, with approximately half of the participants wearing their dentures overnight.

Across the full sample (*n* = 71), the mean number of PFUs was 2.28 (±2.65). However, as stated in Section 2.4, individuals who were edentulous and did not have dental prostheses were excluded from the multivariable modeling (analytical *n* = 65) to prevent artificial floor effects that could bias the inferential estimates.

Clinical assessments revealed an average PI score of 1.83 (±0.65) and an average GI score of 1.19 (±0.68). Subjectively, 17 (30.4%) of the 56 respondents capable of providing a reliable report experienced oral pain in the past 12 months, while xerostomia was assessed in 42 participants with a mean SXI-5 score of 7.38 (±2.18). The mean MNA score was 16.92 (±3.75), placing the average participant in the ‘malnourished’ or ‘risk of malnutrition’ category. Finally, 35 participants (49.3%) consumed food of a normal texture, while 36 participants (50.7%) used a modified texture, including food that was cut, chopped or pureed, or delivered via a nasogastric tube.

The findings from the multivariable models for all oral health outcomes are presented in Table 3A,B. Table 3A addresses cumulative dental disease burden and tooth retention in the full cohort (*N* = 71), and Table 3B covers periodontal status and subjective symptoms. Due to clinical barriers inherent in advanced dementia, analytical samples varied across outcomes, ranging from *n* = 31 for periodontal indices to *n* = 71 for outcomes based on the full clinical examination. For the DMFT outcome, a dual modeling strategy was adopted: an OLS model was used for demographic and clinical predictors, and separate negative binomial (NB) models were used for medication-related factors to ensure the stability of the estimates. Predictor selection for each model was informed by ridge regression, clinical relevance, and statistical parsimony. For example, residence in a long-term care unit (LTCU) was included in the DMFT model as an indicator of accumulated disease over a lifetime, but it was excluded from the periodontal and subjective models to prevent overparameterization in smaller analytical subsets. Detailed information on model specifications is provided in Section 2.4.

#### 3.2.1. Dental Status, Cumulative Oral Disease Burden and Prosthetic Status

Dental status showed wide variability across participants. In the multivariable OLS model including the main demographic and clinical predictors, increasing age and residence in public LTCUs were independently associated with a higher cumulative dental disease burden, as measured by the DMFT index. Each additional year of age was associated with an average increase of 0.48 teeth in the DMFT index (β = 0.48; 95% CI: 0.19 to 0.78; *p* = 0.0017). From a clinical perspective, a 10-year increase in age was associated with approximately 4.8 additional affected teeth. Furthermore, individuals residing in public LTCUs presented, on average, 6.65 more teeth included in the DMFT index than those living in private residences, independently of other factors (β = 6.65; 95% CI: 2.67 to 10.60; *p* = 0.0014). In this main model, MNA and GDS values, sex and polypharmacy were not independently associated with DMFT after multivariable adjustment. The OLS model for the DMFT index demonstrated a statistically significant fit (F (10,60) = 2.40; *p* = 0.018) and explained approximately 16.6% of the variability in the index (Adjusted R^2^ ≈ 0.17).

The distribution of the number of natural teeth was heterogeneous, with participants ranging from edentulism (*n* = 20) to near-complete dentition. The ordinal regression model assessing the number of natural teeth present demonstrated a good overall fit and no evidence of problematic multicollinearity (all VIFs < 2). Male sex was independently associated with a greater number of natural teeth present (OR = 4.35; 95% CI: 1.62 to 11.63; *p* = 0.003). In contrast, a higher number of daily medications was associated with fewer natural teeth: for each additional drug prescribed, the probability of the patient having more teeth decreased by 18% (OR = 0.82; 95% CI: 0.71 to 0.95; *p* = 0.008). Additionally, age showed a borderline inverse association with the number of natural teeth present. GDS and MNA values, toothbrushing habits, and diet texture were not significantly associated with this outcome.

Edentulism status was analyzed using a binary logistic regression model (Table 3). Male sex was significantly associated with lower odds of total edentulism (OR = 0.10; 95% CI: 0.02 to 0.49; *p* = 0.005). No other predictors—including age, polypharmacy, GDS and MNA scores—showed a significant independent association with this outcome (*p* > 0.05). Additional analyses restricted to the edentulous subgroup regarding prostheses use generated imprecise estimates and were considered exploratory due to the small sample size.

#### 3.2.2. Gingival Inflammation and Plaque Indices

Gingival inflammation (GI) and dental plaque accumulation (PI) were analyzed as continuous outcomes using multivariable linear regression models. Each model was based on a final analytical sample of 31 participants due to barriers to clinical assessment in advanced dementia (see Table A1). Both models showed an acceptable overall fit, with no evidence of multicollinearity.

Higher PI values were independently associated with male sex (β = 0.56; 95% CI: 0.15 to 0.98; *p* = 0.010), greater dementia severity (GDS score) (β = 0.22; 95% CI: 0.01 to 0.44; *p* = 0.043), and suboptimal tooth brushing frequency (specifically, several times per week) (β = 0.65; 95% CI: 0.01 to 1.29; *p* = 0.046). Additionally, a modified-texture diet was independently associated with lower PI values (β = −0.64; 95% CI: −1.16 to −0.11; *p* = 0.021). Age and nutritional status (MNA scores) were not independently associated with PI (*p* > 0.05).

Regarding GI, the overall model was significant (F (8, 22) = 2.49; *p* = 0.043); however, none of the individual predictors reached conventional statistical significance after full adjustment. Although not significant, consistent trends were observed: better nutritional status (higher MNA scores) was associated with lower GI levels (β = −0.08, *p* = 0.061), and males tended to exhibit higher GI scores than females (β = 0.47, *p* = 0.070).

Beyond these indices, clinical observation showed that most dentate participants had dental calculus. The prevalence of retained roots was notably high, with several patients presenting multiple infectious foci. Extreme cases recorded as many as 13 or 14 retained roots per arch (*n* = 2).

#### 3.2.3. Oral Pain, Xerostomia and Soft Tissue Alterations

The prevalence of self-reported oral pain was analyzed using binary logistic regression. No clinical, demographic, or behavioral predictors demonstrated statistically robust associations with the presence of pain. The limited number of events and outcome imbalance reduced model stability and should be considered when interpreting these findings.

Xerostomia was analyzed using multivariable linear regression. The final adjusted model showed limited explanatory power, and none of the non-pharmacological predictors—including age, sex, polypharmacy, dementia severity, nutritional status, toothbrushing habits or diet texture—were significantly associated with xerostomia. Similar conclusions were obtained in more parsimonious sensitivity specifications, although these findings should be interpreted cautiously, given the reduced analytical sample and reliance on self-reported symptoms.

Inspection of the soft tissues revealed a prevalence of various oral lesions. Ulcerative lesions were observed on the palate and buccal mucosa of five individuals, often in association with trauma from ill-fitting dentures or fractured teeth. Other benign proliferative lesions, including fibromas (*n* = 4) and mandibular tori (*n* = 2), were described. Lesions requiring specific follow-up were also detected, including probable hemangiomas (*n* = 3) and white lesions, depapillated tongues, fissured tongues, and heavily coated tongues (*n* = 9). These are conditions that affect taste sensitivity and overall comfort during feeding.

#### 3.2.4. Polypharmacy

The study population took an average of 8.9 medications per day (SD = 3.1, range 1–15), indicating a high burden of daily medications. Medication-related predictors were examined separately from the main OLS model for DMFT. For the DMFT outcome, negative binomial regression models were fitted for overall polypharmacy and selected pharmacological classes, and the corresponding estimates were reported as IRRs. In these medication-focused analyses, overall medication count was not significantly associated with DMFT (*p* = 0.229). In contrast, anticoagulant/antiplatelet use showed a non-significant trend toward higher cumulative dental disease (IRR = 1.214, *p* = 0.065).

Multivariable analysis also identified total polypharmacy as an independent, statistically significant predictor of tooth loss, as indicated by a lower likelihood of retaining natural teeth in the ordinal logistic regression model. Each additional daily medication was associated with an 18% decrease in the likelihood of retaining natural teeth (OR = 0.82; 95% CI: 0.71–0.95; *p* = 0.008).

A detailed pharmacological analysis revealed a high prevalence of xerogenic medications, particularly antipsychotics and antidepressants. However, the total drug count did not predict perceived oral dryness (*p* = 0.597). Interestingly, cumulative anticholinergic burden showed a statistically significant yet inverse association with self-reported xerostomia (β = −0.426, *p* = 0.005). This indicates that patients with a higher pharmacological risk unexpectedly reported lower dry mouth severity.

Regarding other pharmacological classes, anticoagulant/antiplatelet use showed a non-significant trend toward higher cumulative dental disease (IRR = 1.214, *p* = 0.065) and increased gingival bleeding (β = 0.232, *p* = 0.117). Calcium-channel blockers were not associated with higher plaque (β = −0.046, *p* = 0.873) or gingival indices (β = −0.087, *p* = 0.749). Finally, diuretics showed no significant associations with any of the primary oral outcomes (all *p* > 0.05).

#### 3.2.5. Nutritional Status

Nutritional status assessed using the MNA revealed that a substantial proportion of participants were either malnourished (MNA < 17) or at risk of malnutrition (MNA = 17–23.5). Nutritional status was not independently associated with most oral health outcomes after multivariable adjustment. Nevertheless, consistent directional trends suggested that better nutritional status was associated with lower levels of gingival inflammation, although these associations did not reach statistical significance.

## 4. Discussion

Patients with dementia are often overlooked in research due to the complexities involved in their recruitment and assessment, both clinical and ethical. However, they are a uniquely fragile group with high comorbidity levels. This study observes a substantial burden of oral disease and functional impairment among institutionalized older adults with dementia. The mean DMFT score of 24.3 reflects a substantial cumulative oral disease burden in this population. This finding aligns with systematic reviews showing poorer oral health in individuals with cognitive decline compared to cognitively intact peers [6,7,8,9,10,11]. Our findings identify two independent factors associated with dental disease burden: older age and residence in public long-term care units (LTCUs). While age-related deterioration is expected—with an average increase of 4.8 affected teeth due to caries or tooth loss for every additional decade of life—an institutional setting suggests a potential link to the care environment itself. On average, participants in public LTCUs had 6.65 more affected teeth than those in private nursing homes, regardless of dementia severity or nutritional status. These results suggest that structural or organizational differences, such as hygiene routines, caregiver training or access to professional dental care, may be associated with variations in oral health outcomes. Although recruitment was restricted to a single institutional network, the network’s dual nature (private residences versus public LTCUs) mitigated potential socioeconomic bias. In Portugal, living in a public LTCU often indicates a lower socioeconomic status, a factor associated with oral health inequalities in Portugal [33]. This cumulative lack of access is reflected in the significantly higher DMFT scores observed among the public LTCU residents in our sample. Furthermore, systematic evidence shows that, although healthcare professionals recognize the importance of oral care, they often find it difficult to provide it due to organizational challenges, such as a lack of time and resources, and a persistent gap between their formal training and daily clinical practice [34]. Regarding tooth retention, the results revealed a significant gender disparity, with males having lower odds of being totally edentulous than females (OR = 0.10, *p* = 0.005). These disparities may reflect underlying differences in health behaviors, access to dental care, or biological resilience.

Polypharmacy was observed to be an independent factor associated with tooth loss; each additional daily medication was associated with an 18% decrease in the likelihood of retaining natural teeth (odds ratio [OR] = 0.82; *p* = 0.008). This finding aligns with evidence that chronic exposure to complex medication regimens, which are common in dementia management, accelerates the progression of dental disease [35]. However, an unexpected inverse association was observed between the anticholinergic burden and self-reported xerostomia (SXI-5) (β = −0.426, *p* = 0.005). Biologically, anticholinergic agents reduce salivary fluid secretion by blocking muscarinic M3 receptors [35,36,37]. Although objective hyposalivation was anticipated, our results suggest that patients with the highest pharmacological risk reported lower dry mouth severity. It is important to distinguish between an objective reduction in salivary flow (quantity), which is typically caused by these medications, and the subjective sensation of dry mouth (xerostomia), which was measured in this study. Furthermore, although the SXI-5 was only administered to a subset of participants (*n* = 42) capable of providing reliable responses, who likely represent individuals with lower cognitive decline than the total sample average, the inverse pattern persisted. One possible explanation for this discrepancy is that cognitive impairment may be associated with anosognosia or communication barriers that affect a patient’s ability to accurately perceive or verbalize physiological symptoms, such as thirst or mucosal dryness, even at these stages. The clinical reality is perhaps better reflected by the extensive dental destruction and high prevalence of retained roots (up to 14 per arch), which contrasts sharply with the lack of verbal complaints. Regarding other medication classes, the use of anticoagulants and antiplatelets showed a non-significant trend toward higher cumulative dental disease burden (IRR = 1.214, *p* = 0.065) and increased gingival bleeding (β = 0.232, *p* = 0.117). Although these results did not reach conventional significance, the direction of the estimates is consistent with the literature regarding drug-induced bleeding risks. Conversely, calcium-channel blockers were not associated with higher plaque (β = −0.046, *p* = 0.873) or gingival indices (β = −0.087, *p* = 0.749) in this cohort. The absence of drug-induced gingival overgrowth may indicate that periodontal status in these patients reflects historical neglect and current assisted hygiene rather than the direct effects of specific medications.

Our results showed that dementia severity (GDS) was associated with increased plaque accumulation (PI). As cognitive and motor decline progress, the ability to maintain adequate oral hygiene may diminish, potentially leading to greater biofilm retention. Intriguingly, our results showed that participants on modified-texture diets had significantly lower plaque levels than those on general diets (β = −0.64, *p* = 0.021). This result is hypothesis-generating, suggesting a potential ‘caregiver paradox’: as dementia progresses to stages of greater functional dependence (GDS 4–7), the need to adapt the diet may be accompanied by stricter institutional surveillance. While patients in early stages may be mistakenly perceived as autonomous and most participants who could respond claimed to be autonomous in their oral hygiene routines, this perception contrasts sharply with their clinical profile: 66.2% of the sample had moderate to severe cognitive impairment (GDS 4–7). Furthermore, while soft diets may be cariogenic, their consistency may facilitate mechanical plaque removal by caregivers, whereas fibrous residues may be retained in patients with decreased motor coordination. Additionally, modified-texture diets were predominantly observed among participants with significant tooth loss. Consequently, the PI may appear lower in some cases due to the reduced number of remaining index teeth available for evaluation. This interplay between assisted hygiene, dietary adaptation, and tooth loss further complicates the clinical management of nutritional risk in advanced neurodegeneration.

The nutritional status of the cohort was concerning, with a mean MNA score of 16.92; a substantial proportion of participants were either malnourished or at risk of malnutrition. Although nutritional status was not a statistically significant independent predictor for most oral health outcomes in our multivariable models, a consistent trend was observed: better nutrition was associated with lower levels of gingival inflammation. These findings align with literature linking impaired masticatory function—evidenced in our cohort by a notably low mean number of PFUs (mean = 2.28)—with compromised dietary intake and increased risk of protein-energy malnutrition [15,16,26,38].

Our multivariable models did not reveal any statistically significant predictors for oral pain. However, this lack of significance should not be interpreted as an absence of pathology; it may instead reflect the severe communication barriers associated with advanced cognitive decline, which affected 66.2% of our sample (GDS 4–7). At these stages, patients often lose the ability to verbalize symptoms and may express orofacial discomfort through behavioral changes. The presence of alarming clinical conditions—such as participants with up to 14 retained roots and active abscesses without verbal complaints—suggests that physical suffering is likely underreported in self-assessment tools in this population.

Most participants demonstrated an immediate or urgent need for treatment according to WHO criteria [23], reflecting the severity of their clinical status. Poor oral health and impaired chewing capacity are factors often associated with precursors to aspiration pneumonia [39] and malnutrition, conditions that can significantly increase caregiver workload and healthcare expenditures [38,40]. In Portugal, hospitalization costs for community-acquired pneumonia are a massive burden on the national health system [41]. Our findings suggest that investing in preventive oral care within nursing homes represents a potentially cost-effective strategy to preserve health and optimize public resources.

### 4.1. Study Limitations

Despite its clinical relevance, this study has limitations that warrant cautious interpretation. First, the cross-sectional design precludes causal inferences, so the findings should be viewed as independent associations and as generating hypotheses. Second, the small overall sample size (*n* = 71), together with reduced analytical samples for specific outcomes—particularly periodontal indices (*n* = 31), xerostomia (*n* = 42), oral pain (*n* = 56), and PFUs (*n* = 65)—limits statistical power and increases the uncertainty of some estimates. These reductions were due to clinical and communication barriers inherent in advanced dementia, as documented in Table A1. Third, the dual-model network (private versus public LTCUs) captured socioeconomic diversity; however, convenience sampling and a complete-case approach may introduce selection bias by excluding more clinically unstable or nonverbal individuals. Fourth, measurement and recall bias cannot be ruled out. Assessments that rely on self-reports (e.g., xerostomia and oral pain) may be affected by anosognosia and communication barriers in patients with advanced dementia. To mitigate this issue, variables dependent on precise memory (e.g., dental history) or perceived need (e.g., hygiene assistance) were excluded from inferential models due to clinical implausibility. Additionally, although medical data (e.g., GDS) was obtained from professional records to ensure accuracy, subjective symptoms were intentionally collected from patients to identify discrepancies between objective pathology and patient perception. Fifth, while the analytical approach is robust and designed to ensure stability in a small cohort, it is complex relative to the sample size. Finally, the absence of a non-dementia comparison group limits our ability to isolate the specific effects of neurodegeneration from the effects of general aging or institutionalization.

### 4.2. Future Research

The findings of this study highlight several critical areas for future research.

- Longitudinal Multicenter Designs: These designs are essential for identifying causal relationships between cumulative pharmacological exposure and the progression of cognitive impairment, as well as the bidirectional link between oral deterioration and nutritional decline over time.
- Objective Salivary Flow Metrics: Prioritizing objective assessments is essential to bypass the communication barriers and anosognosia inherent in advanced dementia.
- Frontline screening tools: The development and validation of simplified clinical screening indices are crucial to empowering caregivers to identify active infectious foci, oral pain and other disorders in nonverbal patients early on.
- Interventional Trials: Research should evaluate the long-term impact of oral hygiene training protocols for caregivers and direct professional dental interventions on preserving oral and nutritional integrity.

## 5. Conclusions

This study observed that institutionalized older adults with dementia face a dual burden of poor oral health and nutritional risk. This burden showed significant associations with advanced age and the institutional setting, particularly in public LTCUs. Polypharmacy emerged as a significant independent factor associated with tooth loss, suggesting that pharmacological management may impact dental health in this population. Regarding the unexpected inverse association between anticholinergic burden and perceived oral dryness, our findings suggest that subjective reports may be unreliable indicators of physiological burden in advanced dementia, likely due to communication barriers associated with cognitive decline. These results suggest that institutional protocols could consider prioritizing objective clinical screenings over exclusive reliance on patient feedback. To bridge the medical–dental gap, we propose exploring the integration of oral health professionals into multidisciplinary teams and implementing regular assessments. Such measures could potentially contribute to preventing costly systemic complications and promoting health in this vulnerable population. However, further longitudinal studies with larger cohorts are necessary to validate these associations and their systemic implications.

## Figures and Tables

**Table 1:** Sociodemographic and Clinical Characteristics of the Study Population (*n* = 71).

Variable	Summary Statistics (*n*, % or Mean ± SD)
Age (years) Mean (SD) [Median; Range]	82.5 (6.9) [83; 59–95]
Gender *n* (%)	
Male	31 (43.7%)
Female	40 (56.3%)
Education Level *n* (%)	
No formal education	8 (11.3%)
Primary Education	43 (60.6%)
Secondary Education	7 (10.0%)
Higher Education	11 (15.5%)
N/A	2 (2.8%)
Marital Status *n* (%)	
Single	8 (11.3%)
Married	31 (43.6%)
Divorced	4 (5.6%)
Widowed	26 (36.6%)
Domestic Partnership	2 (2.1%)
Daily Medications Mean (SD) [Median; Range]	8.9 (3.1) [9; 1–15]
Smoking Status *n* (%)	
Non-smoker	30 (42.3%)
Former smoker	19 (26.8%)
Current smoker	2 (2.8%)
Data not available (N/A)	20 (28.2%)
Global Deterioration Scale (GDS) *n* (%)	
GDS 1: No cognitive decline	0 (0%)
GDS 2: Very mild cognitive decline	5 (7.0%)
GDS 3: Mild cognitive decline	19 (26.8%)
GDS 4: Moderate cognitive decline	15 (21.1%)
GDS 5: Moderately severe decline	13 (18.3%)
GDS 6: Severe cognitive decline	13 (18.3%)
GDS 7: Very severe cognitive decline	6 (8.5%)

SD: Standard Deviation; *n*: sample size; N/A: Not Available; GDS: Global Deterioration Scale.

**Table 2:** Descriptive Summary of Oral Health and Nutritional Data (*n* = 71).

Variable	Summary Statistics (*n*, % or Mean ± SD)
Oral Hygiene Habits, *n* (%)	
Brushing ≥2 times/day	25 (35.2%)
Brushing 1 time/day	27 (38.0%)
Brushing weekly/occasionally	9 (12.7%)
Never/No brushing	10 (14.1%)
Cumulative oral disease burden (DMFT index), mean ± SD	24.31 ± 7.48
Number of Natural Teeth, *n* (%)	
0 teeth	20 (28.2%)
1–9 teeth	18 (25.4%)
10–19 teeth	20 (28.2%)
20–25 teeth	10 (14.1%)
26–32 teeth	3 (4.2%)
Number of retained roots, mean ± SD	1.42 ± 2.84 *
Prosthetic Status, *n* (%)	
Non-edentulous with prostheses	15 (21.1%)
Non-edentulous without prostheses	36 (50.7%)
Edentulous with prostheses	14 (19.7%)
Edentulous without prostheses	6 (8.5%)
Oral Hygiene & Inflammation Indices	
Plaque Index (PI), Mean ± SD	1.83 ± 0.65
Gingival Index (GI), Mean ± SD	1.19 ± 0.68
Reported Oral Pain, *n* (%) (*n* = 56)	17 (30.4%)
Xerostomia (SXI-5) (*n* = 42), mean ± SD	7.38 ± 2.18
Posterior Functional Units (PFUs) (*n* = 65), mean ± SD	2.28 ± 2.65
Nutritional Status (MNA score), mean ± SD	16.92 ± 3.75
Malnourished (<17 points), *n* (%)	36 (50.7%)
At risk of malnutrition (17–23.5 points), *n* (%)	34 (47.8%)
Normal nutritional status (24–30 points), *n* (%)	1 (1.4%)
Diet Texture Type, *n* (%)	
Normal	35 (49.3%)
Cut	19 (26.8%)
Paste	13 (18.3%)
Chopped	2 (2.8%)
Nasogastric tube	2 (2.8%)

SD: Standard Deviation; *n*: sample size; * retained roots mean value was calculated based on the full sample (*n* = 71), although only 28 individuals presented at least one retained root (39.4%).

**Table 3:** (**A**) Multivariable Analysis of Dental Outcomes. (**B**) Multivariable Analysis of Soft Tissue, Periodontal and Subjective Outcomes.

(**A**)					
**Predictor Variable**	**DMFT** (OLS β/NB IRR *)	**Natural Teeth** **(OR)**	**Total Edentulism** **(OR)**	**PFUs** (IRR) **	**Retained Roots** **(IRR)**
Male sex	1.17 (0.555)	**4.35 (0.003)**	**0.10 (0.005)**	1.34 (0.355)	-
Age (years)	**0.48 (0.002)**	0.94 (0.079)	1.07 (0.218)	1.00 (0.928)	-
Residence: public LTCU	**6.65 (0.001)**	-	-	-	-
Dementia (GDS)	0.62 (0.480)	0.94 (0.753)	1.12 (0.705)	0.85 (0.193)	-
MNA score	0.36 (0.251)	0.96 (0.628)	1.00 (0.989)	0.94 (0.144)	1.00 (0.994)
Modified Diet	-	0.61 (0.356)	1.99 (0.365)	0.85 (0.627)	-
Polypharmacy (count)	0.35 (0.229)	**0.82 (0.008)**	1.17 (0.124)	0.97 (0.420)	-
Anticholinergic Burden	1.02 (0.503)	-	-	-	1.03 (0.785)
(**B**)					
**Predictor Variable**		**PI (β)**	**GI (β)**	**Oral Pain (OR)**	**SXI-5 (β)**
Male sex		**0.56 (0.010)**	0.47 (0.070)	1.89 (0.342)	−0.38 (0.599)
Age (years)		−0.00 (0.909)	0.01 (0.750)	1.02 (0.693)	−0.06 (0.274)
Dementia (GDS)		**0.22 (0.043)**	0.04 (0.707)	0.90 (0.687)	−0.27 (0.384)
MNA score		−0.05 (0.219)	−0.08 (0.061)	0.96 (0.707)	−0.09 (0.458)
Modified Diet ***		**−0.64 (0.021)**	−0.03 (0.933)	0.96 (0.954)	0.65 (0.450)
Polypharmacy		-	−0.07 (0.101)	0.89 (0.229)	−0.06 (0.597)
Anticholinergic Burden		0.03 (0.529)	0.06 (0.251)	-	**−0.43 (0.005)**

DMFT: Decayed, Missing, Filled Teeth; OLS: Ordinary Least Squares; β: Unstandardized regression coefficient; OR: Odds ratio; IRR: Incidence rate ratio; LTCU: Long-term care unit; GDS: Global Deterioration Scale; MNA: Mini Nutritional Assessment; PFUs: Posterior Functional Units; PI: Plaque Index; GI: Gingival Index; SXI-5: Short Xerostomia Inventory; * for the DMFT outcome, estimates for demographic and clinical predictors derive from the multivariable OLS model and are reported as unstandardized β coefficients. Medication-related predictors were examined in separate negative binomial models and are reported as IRRs. Because these estimates derive from different model families, they should not be directly compared within the same column. ** PFUs were calculated for participants with natural teeth or dental prostheses (*N* = 65) to avoid artificial floor effects. *** a modified diet includes textures such as cut, chopped, or paste, as well as nasogastric tube feeding. Predictors were selected based on clinical plausibility and penalized regression (ridge) to ensure stability in this sample (*N* = 71). Analytical samples vary across outcomes (*n* = 31 for PI and GI; *n* = 56 for oral pain; *n* = 42 for SXI-5). See Table A1 for detailed exclusion reasons. Statistically significant results (*p* < 0.05) are in **bold**.

## Data Availability

The data presented in this study are available on request from the corresponding author. The data are not publicly available due to privacy and ethical restrictions.

## Abbreviations

The following abbreviations are used in this manuscript:

DMFT	Decayed, Missing, Filled Teeth
GDS	Global Deterioration Scale
GI	Gingival Index
IRR	Incidence Rate Ratio
LTCU	Long-Term Care Unit
MNA	Mini Nutritional Assessment
MUAC	Mid-Upper Arm Circumference
OR	Odds Ratio
PEG	Percutaneous Endoscopic Gastrostomy
PFU	Posterior Functional Unit
PI	Plaque Index
PPI	Proton Pump Inhibitor
SD	Standard Deviation
STROBE	Strengthening the Reporting of Observational Studies in Epidemiology
SXI-5	Short Xerostomia Inventory
WHO	World Health Organization
ZINB	Zero-Inflated Negative Binomial

## Appendix A

**Table A1:** Summary of analytical samples and reasons for missing data across study outcomes.

Outcome/Variable Analytical	Sample (*n*)	Primary Reasons for Missingness/Exclusion
Retained Roots	71	Full clinical sample evaluated during intra-oral examination.
Decayed Missing Filled Teeth (DMFT) index	71	Full clinical sample evaluated during intra-oral examination.
Oral Pain	56	Non-verbal status, bedridden condition, incoherent speech.
Xerostomia (SXI-5)	42	Significant cognitive impairment preventing reliable questionnaire responses.
Plaque Index (PI)	31	Absence of index teeth (total edentulism) or extreme lack of cooperation (e.g., biting instruments).
Gingival Index (GI)	31	Absence of index teeth (total edentulism) or extreme lack of cooperation (e.g., biting instruments).
Posterior Functional Units (PFUs)	65	Exclusion of edentulous individuals without prostheses.
